# Myxedema coma precipitated by diabetic ketoacidosis after total thyroidectomy: a case report

**DOI:** 10.1186/s13256-019-1992-0

**Published:** 2019-03-04

**Authors:** Jin Joo Kim, Eun Young Kim

**Affiliations:** Division of Trauma and Surgical Critical Care, Department of Surgery, Seoul St. Mary’s Hospital, College of Medicine, The Catholic University of Korea, Banpo-daero 222, Seocho-gu, Seoul, 137-701 Korea

**Keywords:** Myxedema coma, Diabetic ketoacidosis, Total thyroidectomy, Hypothyroidism

## Abstract

**Background:**

Myxedema coma is profound decompensated hypothyroidism usually precipitated by stressors, and its occurrence in association with total thyroidectomy or metabolic disorders, such as diabetic ketoacidosis, is unusual.

**Case presentation:**

A 43-year-old Asian man with history of total thyroidectomy who was scheduled for a second radioactive iodine therapy presented to our hospital with decreased mental status and hyperglycemia. He had a history of thyroid cancer but did not have diabetes mellitus. He was in a hypothermic state and had a Glasgow Coma Scale score of 10 out of 15 at presentation; arterial blood gas analysis revealed a state of metabolic acidosis and laboratory findings suggested hyperglycemia with glycosuria, ketoacidosis, and severe hypothyroidism. A thyroid function test showed thyroid-stimulating hormone of 34.126 uIU/mL, free thyroxine of 1.02 ng/dL, and triiodothyronine of 1.04 ng/mL. The glycated hemoglobin of this patient was checked due to hyperglycemia and the value of glycated hemoglobin was 16.5% which met the criteria for a diagnosis of diabetes. After treatment for myxedema with liothyronine 5 mcg two times per day and levothyroxine 175 mcg once daily via a nasogastric tube and diabetic ketoacidosis with intravenously administered fluid and insulin, his clinical condition rapidly improved including mental status, hyperglycemia, and acidosis. During the hospitalization, a workup for diabetes mellitus was performed and the results suggested that a diagnosis of type 2 diabetes mellitus would be appropriate.

**Conclusions:**

This case demonstrated that diabetic ketoacidosis not only could be a potential contributor to myxedema coma but also mask typical clinical features, making diagnosis more difficult. Considering the possibility of an increasing number of potential patients with hypothyroidism developed after thyroidectomy, constant vigilance is required for a better clinical outcome, including early recognition and management in critical care in advance for unusual diabetic ketoacidosis which could precipitate decompensated hypothyroidism.

## Background

Myxedema coma is one of the potentially lethal complications of hypothyroidism, and the term might account for profound decompensated hypothyroidism caused by a stressor such as infection. Although myxedema coma is rare, the mortality rate has been reported to be high: approximately 50–60% in recent studies [1, 2]. Early recognition and more aggressive management have been noted to be important because they are expected to reduce the mortality rate to closer to 20–25% [3].

Myxedema coma usually seems to be more common in older women during the winter period; it is also known to be precipitated by environmental cold exposure, infection, sepsis, stroke, cardiovascular compromise, or medication. There are several clinical features including hypothermia, altered mental status, bradycardia, hypotension, hypercapnia, or delayed deep tendon reflexes. An electrocardiogram shows QT prolongation and sinus bradycardia in most cases, and gastric atony or paralytic ileus can be seen from plain X-ray. The diagnosis of myxedema coma can be made with a patient who has a history of thyroid disease or thyroidectomy with altered mental status or hypothermia. However, it can be misdiagnosed due to various clinical conditions that can present several manifestations similar to those of myxedema coma, such as sepsis, heart failure, cerebrovascular disease, drug use, or metabolic disorder [4]. Here we report the case of a 43-year-old man with undiagnosed diabetes mellitus who showed atypical clinical presentation of myxedema coma precipitated by diabetic ketoacidosis (DKA). Study approvals were obtained from the Institutional Review Board (IRB) of Seoul St. Mary’s Hospital (No. IRB; KC18ZESI0785).

## Case presentation

A 43-year-old Asian man presented to the emergency department in our institution due to generalized weakness in April 2018. One month prior to admission, his family noted that he showed poor oral intake and consistently complained of epigastric discomfort. He was diagnosed as having impaired fasting glucose and hyperlipidemia at the age of 42 on routine medical checkup. Eight months ago, he underwent total thyroidectomy with both central and sentinel lymph node dissection due to papillary thyroid carcinoma and the pathologic stage was diagnosed as T3N1bM0 on the permanent pathologic report. After that, the first radioactive iodine (RAI) therapy was conducted and an iodine [1–3] whole body scan was planned to determine whether to perform the second RAI that was on the next day of visiting the emergency room, therefore, he had to stop the thyroid medication for 3 weeks to prepare for the examination.

**Fig. 1:**
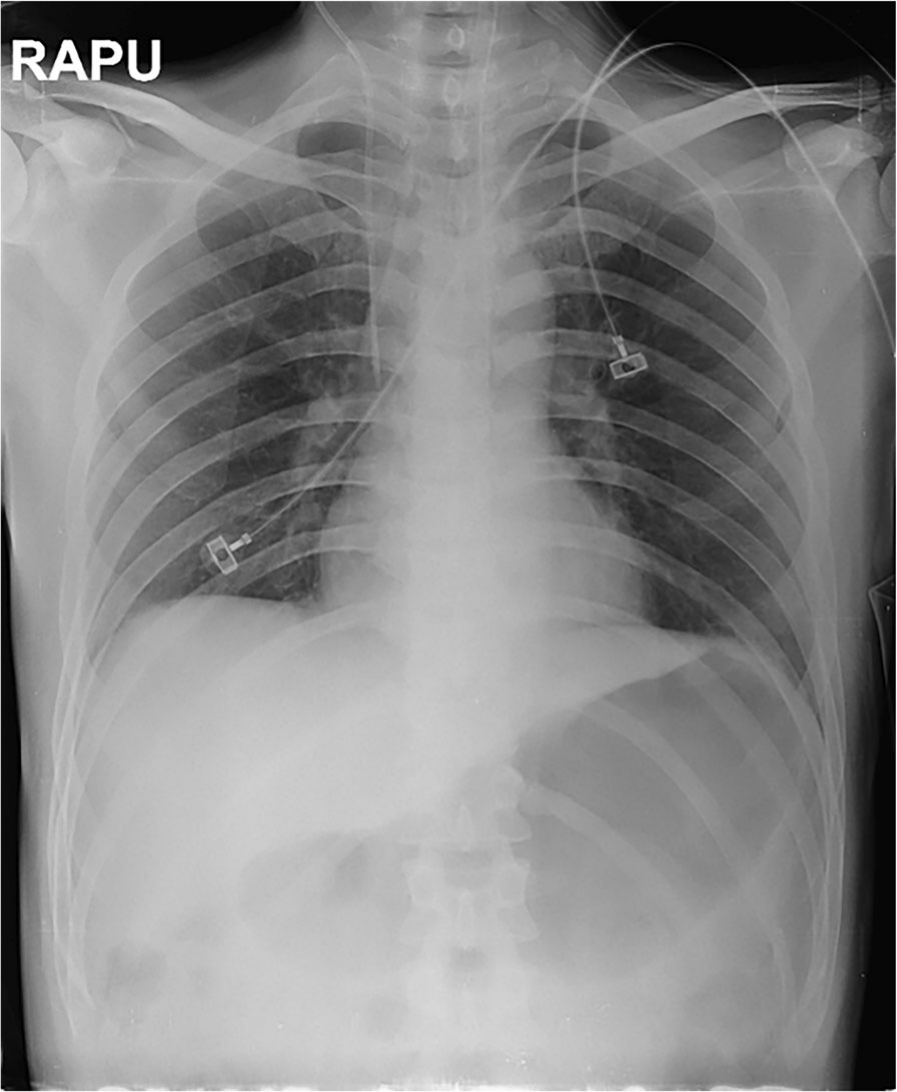
Chest X-ray at presentation

**Fig. 2:**
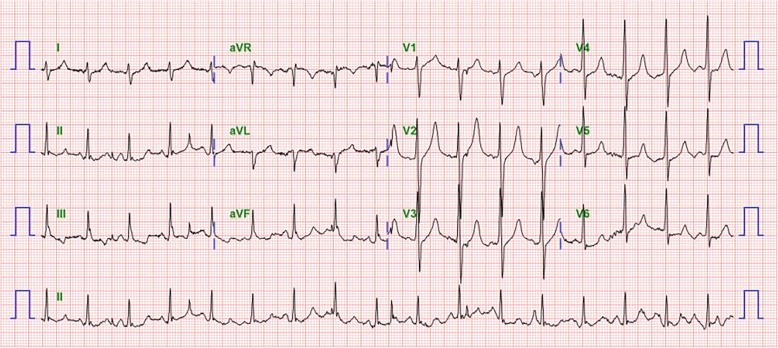
Electrocardiogram at presentation

At the time of admission to the emergency room, he was noted to be somnolent and had a decreased level of consciousness. He opened eyes to pain, showed inappropriate verbal response and flexion withdrawal from pain, which suggested that Glasgow Coma Scale (GCS) was 10 out of 15. On physical examination, there was no pretibial edema and his pupils were equal in size and normally reactive to light. His abdomen was slightly distended with decreased bowel sound and his extremities were cold. His blood pressure was 127/96 mmHg, heart rate was 101 beats per a minute, and respiratory rate was 25 breaths per a minute with oxygen saturation 97% on room air. He was in a hypothermic state and his tympanic temperature was approximately 34.0 °C. Chest radiography revealed the findings of gastroparesis and paralytic ileus as presented in Fig. 1. An electrocardiogram at presentation showed sinus tachycardia with QT prolongation by 537 ms of corrected QT interval (Fig. 2).

Arterial blood gas analysis revealed a state of metabolic acidosis: a pH of 7.00, partial pressure of carbon dioxide in arterial blood (PaCO_2_) of less than 10 mmHg, bicarbonate (HCO_3_) of less than 10 mmol/L, and base excess of − 34.6. Laboratory findings suggested hyperglycemia with glycosuria and ketoacidosis, which are presented in Table 1. Considering the history of total thyroidectomy and planned schedule for RAI, a thyroid function test (TFT) was conducted and revealed severe hypothyroidism. He was found to have a thyroid-stimulating hormone (TSH) of 34.126 uIU/mL (0.55–4.78 uIU/mL) and free thyroxine (T4) of less than 0.01 ng/dL (0.82–1.76 ng/dL) and triiodothyronine (T3) of less than 0.01 ng/mL (0.6–1.81 ng/mL). Even though he did not have any history of diabetes mellitus, we checked his glycated hemoglobin (HbA1c) due to hyperglycemia. Finally, the value of HbA1c was 16.5% which met the criteria for a diagnosis of diabetes.

**Table 1:** Laboratory investigations at admission to the Intensive care Unit

	Values	Reference range
WBC (× 10^9^/L)	25.97	4.0–10.0
Hb (g/dl)	16.8	13.0–18.0
PLT (× 103/L)	395	130–400
Serum glucose (mg/dl)	1020	50–100
Urine glucose	3 positive	Negative
Creatinine (mg/dl)	2.68	0.6–1.2
Serum osmolarity (mosm/kg)	386	28–120
Serum sodium (mEq/L)	138	136–146
Serum potassium (mEq/L)	5.9	3.5–5.1
Serum chloride (mEq/L)	84	98–106
Serum total ketone bodies (umol/L)	> 1000	20–120
Urine ketone	3 positive	Negative

*Hb* hemoglobin, *PLT* platelets, *WBC* white blood cells

**Fig. 3:**
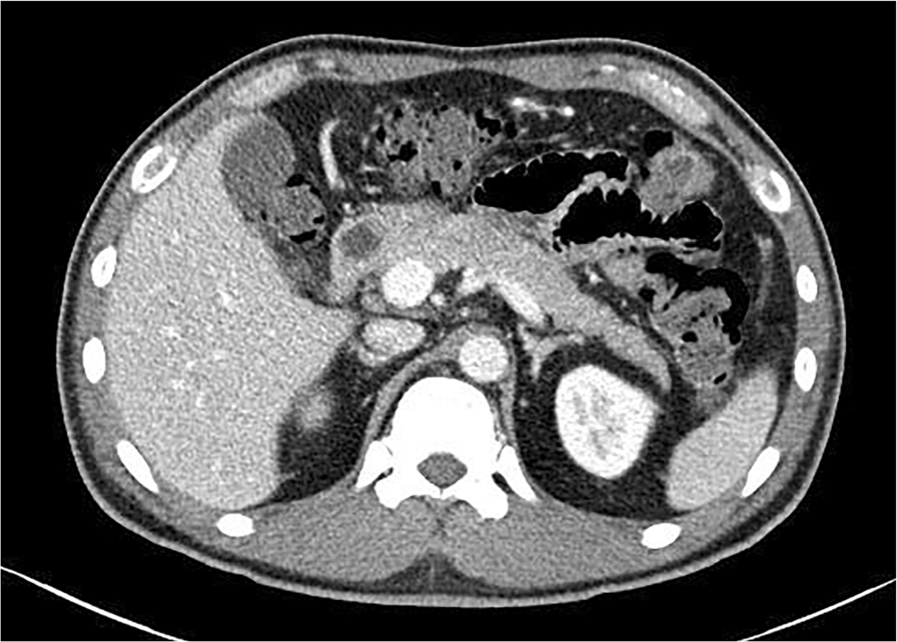
Computed tomography of pancreas. There was no evidence of pancreas mass or pancreatitis

He was admitted to the intensive care unit (ICU) for the management of DKA and myxedema coma. He received intravenously administered fluid with electrolytes and an immediately applied insulin pump. For hormonal replacement, liothyronine 5 mcg two times per day and levothyroxine 175 mcg once daily were administered via a nasogastric tube. He instantly responded to the therapy with a favorable clinical improvement. His mental status started to improve several hours after treatment and at the third day of hospitalization he showed a GCS of 15/15; his body temperature increased from 34 °C to 36.5 °C approximately 10 hours after admission. The metabolic acidosis was corrected 6 hours after administration of intravenously administered fluid with insulin pump and hyperglycemia was also improved; the insulin pump was discontinued then and switched to subcutaneous insulin 1 day after hospitalization. Repeated TFT before discharge revealed TSH of 21.798 uIU/mL (0.55–4.78 uIU/mL), free T4 of 1.02 ng/dL (0.82–1.76 ng/dL), and T3 of 1.04 ng/dL (0.6–1.81 ng/mL). The clinical course of this patient was summarized in Table 2. During the hospitalization, a workup for diabetes mellitus was performed and there was no evidence of pancreas mass or pancreatitis on abdominal computed tomography (Fig. 3). Results from investigations for diabetes mellitus showed a fasting c-peptide of 1.08 ng/mL (0.48–3.30 ng/mL), anti-islet cell antibodies (ab) negative, and glutamic acid decarboxylase (GAD) ab of 0.01 U/ml which suggested that a diagnosis of type 2 diabetes mellitus would be appropriate.

**Table 2:** Clinical course during hospitalization

	Glucose mg/L	AG nmol/L	Na mEq/L	K mEq/L	Cl mEq/L	Osm mosm/L	TSH uII/mL	Free T4 ng/dL	T3 ng/dL	Neurologic status	
Admission	1020	> 44	138	5.9	84	386	34.126	< 0.01	< 0.01	GCS 10/15	Insulin pump IVF Liothyronine Levothyroxine
HD #2	306	19.5	149	4.0	110	331				GCS 14/15	
HD #3	278	11.45	143	3.6	108	308	41.399	0.19	< 0.01	GCS 15/15	Insulin SQ
Discharge (HD #11)	145		138	4.5	101	296	21.798	1.02	1.04		

*AG* anion gap, *GCS* Glasgow Coma Scale, *HD* hospital day, *IVF* intravenously administered fluid, *Osm* calculated serum osmolarity, *SQ* subcutaneous, *T3* triiodothyronine, *T4* thyroxine, *TSH* thyroid-stimulating hormone

He was discharged from surgical ICU after 2 days, stayed for a further 8 days on the general ward and was discharged on the 11th hospital day with tolerable status. The dose of thyroid hormone medications was subsequently reduced at our out-patient clinic after he was discharged and an endocrinologist recommend insulin with orally administered hypoglycemic agents.

## Discussion and conclusions

Myxedema coma is a medical status in which there is a complex manifestation of decompensated hypothyroidism with hypothermia, hypotension, or decreased level of consciousness. It is known to be a true medical emergency; although it is rare, the overall mortality was reported to be 50-60% in the past. Most cases occur in older women during winter periods; it can be associated with signs of hypothyroidism. A high degree of suspicion is necessary for diagnosis in patients presenting with altered level of consciousness who have a history of thyroidectomy, hypothyroidism, RAI therapy, or a discontinuation or improper administration of thyroid medications because early recognition and intensive supportive care in advance have reduced the mortality rate to 20–25% [2, 3]. *Kumar et al.* [5] previously reported rapid clinical improvement of hemodynamics and respiratory status after thyroid hormone administration in patients with hypothyroid state superimposed on critical illness who did not have underlying thyroid disease. However, careful individual assessment would be needed considering all possible clinical conditions to interpret the result of TFT for critically ill patients. And, regarding causation of our case, we believe that myxedema coma could be a more suitable conclusion.

Previous studies have suggested several poor prognostic factors for myxedema coma and Jordan [6] suggested that old age, bradycardia less than 44 beats per minute, a low serum level of T3, hypothermia of less than 34 °C, and persistent hypotension are risk factors for poor prognosis of myxedema coma. Sepsis and myocardial infarction, and delayed treatment could also affect a poor prognosis. Rodriguez *et al.* [3] also observed that an Acute Physiology and Chronic Health Evaluation (APACHE) II score of more than 20 and the degree of consciousness could determine the prognosis of myxedema coma and it could be precipitated by infection, cardiac events, medications such as antiepileptic drugs, trauma, surgical stress, or exposure to cold temperature. However, few cases have been reported in which myxedema coma could be precipitated by metabolic disorders such as DKA or hyperglycemic hyperosmolar state (HHS) which could also show hyperglycemia, altered mental status, or hypotension [1, 7]. In this patient, DKA would have led to rapid development of myxedema coma albeit he had not been diagnosed as having diabetes mellitus. We believe that DKA could act as a major contributor to the development of myxedema coma, as our patient did not have a period of a long-standing hypothyroidism. In this case, the patient did not have other significant risk factors for myxedema coma such as myocardial infarction or history of medication use except thyroid hormone at presentation.

To the best of our knowledge, there are no reports in the English literature about a case of myxedema coma developing after withdrawal of hormone replacement therapy among patients after thyroidectomy for thyroid cancer or those who are scheduled for RAI therapy. In addition, myxedema coma precipitated by DKA has been described in only two cases in the English literature: one case was precipitated by DKA with neuroleptic drugs, in the other case it was believed that myxedema coma was precipitated by DKA only. However, from both cases, the patients did not have a history of surgical resection of thyroid due to thyroid cancer [4, 8]. Our case is an unusual case of myxedema coma because of the middle age of our patient, absence of history of cold exposure, and having atypical clinical features. It would be unlikely that withdrawal of 40 mg daily of liothyronine for a few weeks could precipitate a myxedema coma, even with an associated DKA [8]. The hallmark of myxedema is alteration of mental status such as lethargy or coma and hypothermia. The hypothermia could be the clinical clue for diagnosis. This patient showed hypothermia with decreased GCS; however, our patient also had atypical features of myxedema such as normal respiratory rate, normal heart rate, and normal blood pressure.

Myxedema would also be typically associated with hyponatremia, hypo-osmolality, and hypoglycemia because of increased antidiuretic hormone production as a result of decreased cardiac output, and reduced renal plasma flow, which could increase total body water [7]. In our case, plasma osmolality was increased with normonatremia which were fairly mild for DKA. It could be possible that coexistence with myxedema may improve the hyperosmolar state of DKA. Although our patient had not been diagnosed previously for diabetes mellitus, he had marked hyperglycemia with high anion gap metabolic acidosis, and significant blood and urine ketones which implied DKA. Moreover, a diagnostic workup for hyperglycemia suggested that a diagnosis of type 2 diabetes mellitus would be appropriate. Actually, DKA was usually regarded to occur only in patients with type 1 diabetes mellitus, however, a retrospective study recently found that among adult patients with DKA, 47% were classified as type 1 diabetes, 26% had known type 2 diabetes, and 27% had DKA as the initial presentation of the diabetes [9]. We suppose that this point would be an impressive clinical manifestation of this patient compared with patients who have an ordinary case of DKA.

The interesting viewpoint of our case includes unusual clinical manifestations, the absence of long-standing hypothyroidism, no history of cold exposure, and the masking of typical clinical features by DKA. We suppose that the diabetes of the patient, who was healthy despite having risk factors for diabetes, was expressed in the form DKA which was triggered by transient severe decompensated hypothyroidism during the RAI process. The incidence of thyroid cancer has increased worldwide and the treatment of thyroid cancer is thyroidectomy followed by RAI [10]. In 2012, approximately two thirds of patients with thyroid cancer underwent radical thyroidectomy and approximately 60% of them received RAI in South Korea [9, 11]. In addition, the prevalence of diabetes mellitus has increased in South Korea and it has been shown that the increased incidence of the lifestyle-related disease is associated with obesity or decreased physical activity, which is a result of urbanization or a change of diet as the undiagnosed risk factors [12]. Therefore, we expect that, similar to this case, the risk of development of a medically critical illness such as myxedema coma precipitated by DKA will be relatively high in patients with unrecognized diabetes and consideration of this clinical condition will be needed.

In conclusion, early recognition of atypical myxedema coma, management in critical care in advance, and timely administration of thyroid hormone therapy are important for better clinical outcome of myxedema coma. Our case demonstrated that DKA not only could be a potential contributor to myxedema coma but also mask typical clinical features, making diagnosis more difficult. Considering the possibility of an increasing number of potential patients with hypothyroidism developed after thyroidectomy, constant vigilance is necessary for unusual DKA which could precipitate decompensated hypothyroidism.

## Abbreviations

Ab: Antibodies
APACHE: Acute Physiology and Chronic Health Evaluation
DKA: Diabetic ketoacidosis
DM: Diabetes mellitus
GAD: Glutamic acid decarboxylase
GCS: Glasgow Coma Scale
HbA1c: Glycated hemoglobin
HCO_3_: Bicarbonate
HHS: Hyperglycemic hyperosmolar state
ICU: Intensive care unit
IRB: Institutional Review Board
PaCO_2_: Partial pressure of carbon dioxide in arterial blood
RAI: Radioactive iodine
T3: Triiodothyronine
T4: Thyroxine
TFT: Thyroid function test
TSH: Thyroid-stimulating hormone
